# Effects of Integrated Indirect Forest Experience on Emotion, Fatigue, Stress, and Immune Function in Hemodialysis Patients

**DOI:** 10.3390/ijerph18041701

**Published:** 2021-02-10

**Authors:** Hyoyoung Kang, Youngran Chae

**Affiliations:** Department of Nursing, Kangwon National University, Chuncheon 24341, Korea; sissy2@naver.com

**Keywords:** hemodialysis, indirect forest experience, emotion, fatigue, stress, heart rate variability, natural killer cells

## Abstract

Background: Most hemodialysis patients may experience physiological and psychological stress. Exposure to nature has been reported to reduce psychological and physiological stress levels and improve immune function. This study aimed to investigate psychological and physiological effects of integrated indirect forest experience on chronic renal failure patients undergoing hemodialysis. Methods: As a quasi-experiment, this study employed a nonequivalent control group, repeated measurements, and a non-synchronized design. In total, 54 participants were included: 26 and 28 patients in the experimental and control groups, respectively. During hemodialysis, five types of forest therapy stimuli (visual, auditory, olfactory, tactile, and motor) were applied 3 times per week for 4 weeks during 15 min sessions. Results: Positive, but not negative, emotion measures differed between the groups after the intervention. Fatigue and physiological stress levels were significantly reduced in the experimental group, whereas no significant difference was found between the groups with respect to measures of psychological stress. Activation of both the parasympathetic and sympathetic nervous systems was similar in both groups, as was the number of natural killer cells. Conclusion: Integrated indirect forest experience may help increase positive emotions and reduce fatigue and stress levels during hemodialysis in patients with chronic renal failure.

## 1. Introduction

Chronic renal failure is an irreversible disease associated with gradual loss of kidney function [1]. In Korea, affected patients tend to choose hemodialysis (81,760 (75.1%) patients), rather than renal transplant or peritoneal dialysis [2]. Without a transplant, most hemodialysis patients continue to undergo dialysis for over 4–5 h, 3–4 times weekly, for the rest of their life [3].

During hemodialysis, patients may experience physiological and psychological stress due to restrictions that prevent them from maintaining their daily routines [4]. The primary factor that causes stress in patients is fatigue [5]. Seventy-nine percent of hemodialysis participants complain of fatigue [6], which tends to increase after hemodialysis [7]. Concurrently, psychological stress in hemodialysis patients tends to be caused by tensions provoked by dependence on hemodialysis machines [8], loss of pride, uncertainty about the future, and feelings of guilt toward family [9], among others. Indeed, previous studies have shown that stress levels in hemodialysis patients are as high as those in patients with terminal cancer [10]. Severe and prolonged stress response may lead to the suppression of the immune system function [11]. Therefore, it is critical to develop stress management interventions for hemodialysis patients.

Stress reduction theory has been proposed by Ulrich: it aims to explain how the natural environment reduces the human response to mental and physical stress, for example, during the sequential process of viewing nature scenes, which may influence one’s emotions and behavior [12]. According to this theory, an individual exposed to visual stimuli associated with the natural environment is likely to experience immediate and general emotional and automatic behavioral response [12]. For example, if the observer is stressed, a view of nature can attract attention and block or reduce stressful thoughts, thus promoting psychophysiological recovery [13]. In line with this theory, forest therapy has been attracting attention, and some studies suggest that it promotes physical health and may strengthen the immune system [14], while others show that it may improve mental and physical health [15], affecting physiological and psychological parameters. Managed by the autonomic nervous system, heart rate variability (HRV) is a biomarker of stress [16,17,18]. Previous studies have indicated that forest therapy may activate the parasympathetic and inhibit the sympathetic nervous system responses [19,20]; moreover, it has also been shown to affect immune function [21], specifically, to increase the activity of natural killer cells (NK) [22].

Indirect forest experience involves providing forest-like stimuli to patients indoors [23]. Psychological and physiological stress levels have been shown to alleviate in response to just viewing nature scenes [24,25]; however, patients tend to prefer receiving both visual and auditory stimuli at the same time (e.g., seeing a nature view while listening to water sounds) [26]. Moreover, olfactory stimulation with wood-scented oil significantly increases parasympathetic activity [27]. Although few studies exist on tactile stimulation, touching wood with feet has been shown to have a positive impact on brain activity and to promote physiological relaxation [28]. In a study that applied an electric leg exercise machine to patients undergoing hemodialysis in a supine position, the patients’ mental health index score increased significantly, which suggests that this could be a safe and effective intervention [29]. 

Recent studies of indirect forest experience mostly focused on healthy individuals [15,20,23], and few of such studies have been performed in a clinical context [30,31]. Studies of indirect forest experience that involve five senses are rare, despite evidence that this approach is more effective than that involving only one or two senses [32]. In fact, in a study of older adults, Park [33] reported that stimulating five senses might be more effective than stimulating only one or two of the senses in indirect forest therapy.

Hemodialysis patients have restricted access to nature due to their treatment and deconditioning schedule. Exposing patients to nature-associated sensory stimuli while they remain in a hospital environment may provide them with physiological and psychological benefits of forest therapy. This study aimed to verify the effects of integrated indirect forest experience on the measures of emotion, fatigue, and stress, HRV, and NK cells count in patients with chronic renal failure undergoing hemodialysis.

## 2. Materials and Methods

### 2.1. Participants

In this quasi-experimental study with a nonequivalent control group, repeated measurements, and a non-synchronized design, 130 patients with chronic renal failure undergoing hemodialysis at a designated unit of a medical clinic were selected using convenience sampling. 

Using repeated measures analysis of variance (ANOVA) with G*Power 3.1 software [34], we determined that 52 individuals in total (26 per group) were required for the present study, given a significance level of 0.05, an effect size of 0.18 [35], and a statistical power of 0.80. Accounting for possible dropouts, we included 64 (32 per group) participants. In the experimental group, 2 participants changed clinics, 1 withdrew, and 3 failed to meet the compliance rate of the experimental group, resulting in the final group size of 26. In the control group, 1 participant changed clinics, 1 experienced health deterioration, and 2 missed hemodialysis sessions, resulting in the final group size of 28. Overall, 54 participants were included in the study, and the dropout rate was 15.6%.

### 2.2. Materials

Demographic and clinical characteristics of interest included age, sex, marital status, education level, occupation, religion, cause of disease, period of hemodialysis, vascular state for hemodialysis, hemodialysis vessel location, hemoglobin, and albumin.

Emotion: The inventory of personal reactions developed by Zuckerman (ZIPERS) [36] was used to examine emotional reactions toward indirect forest environments. The scale included four items of positive affect, two items of attentiveness, with one item being a negative question to ensure faithful answers, three items of fear, one item of sadness, and two items of anger, totaling to five categories and twelve items (Table 1). Among the 12 items of ZIPERS, items #1 to #6 represented positive emotions and items #7 to #12 represented negative emotions [37]. Each question was measured using a 5-point scale (not at all: 0 points, very true: 5 points). The total score ranged from 0 to 30 points, with higher scores indicating that the individual had more positive and negative emotions. In our study, Cronbach’s alpha values were 0.62 and 0.90 for the positive and negative emotion components respectively, with previously reported values ranging from 0.80 to 0.85 [36].

Fatigue: Fatigue was measured using a tool proposed by Lee et al. [38], and it was revised and updated by Kim [39]. Among 17 items, a single item was eliminated [40] as it was not suitable for the present study. This tool comprised a 12-item fatigue subscale (tired, sleepy, dull, drowsy, fatigued, worn out, moving my body, concentrating, carrying on a conversation, desire to lie down, exhausted, and keeping my eyes open) and a 4-item energy subscale (energetic, efficient, vigorous, and active). The total score ranged from 0 (not fatigued at all) to 160 (extremely fatigued) points, measured on a 10-point Likert scale. In our study, Cronbach’s alpha was 0.83, while the previously reported values ranged from 0.94 to 0.96 [38].

Stress: Stress levels were measured using a tool developed for hemodialysis patients by Kim [41], and it was revised and updated by Choi [42]. The tool includes 20 items in total, with physiological and psychological stress interrogated by a set of 10 questions each. Physiological stress included stress from physical symptoms such as muscle cramps, fatigue, skin itching, joint discomfort, sleep disturbances, and decreased concentration and memory. Psychological stress included stress caused by uncertain future, changes in appearance, discomfort in daily life due to hemodialysis, difficulties in work life, economic burden, feeling guilty about the roles of family members in care, and anxiety during hemodialysis. Using 4-point Likert scales, the stress scores yielded by the tool ranged from 10 (not serious at all) to 40 (extremely serious) points, with higher scores indicating that the individual was more stressed. In our study, Cronbach’s alpha was 0.86 for both physiological and psychological stress levels. In Choi’s study, Cronbach’s alpha for physiological stress and psychological stress was 0.86 and 0.79, respectively [42].

To assess the physiological indicator of stress, we assessed minimum changes in heart rate using an HRV meter (Wise-8000T, MooYoo Instrument, Seongnam, Korea), and autonomic nerve responses were observed. HRV measured parameters such as sympathetic nervous system activity (low frequency) and parasympathetic nervous activity (high frequency). The normal range of the sympathetic and parasympathetic nervous system activity is within low (0.04–0.15 Hz) and high (0.15–0.4 Hz) frequency values, with the corresponding power spectrum of 5.9–8.0 and 3.8–7.0, respectively. The examination of HRV was performed during the dialysis session and after using the electric exercise machine.

NK cells: The immune function was measured using flow cytometry to assess the NK cell (CD16 + CD56) levels. To this end, 3 mL of blood was collected in ethylenediaminetetraacetic acid (EDTA) tubes and sent to the Seegene medical foundation laboratory. The instruments for blood sampling (blood collection syringes, sampling tubes, etc.) were disposable.

### 2.3. Indirect Forest Stimulation

We used visual, auditory, olfactory, tactile, and motor stimulation for all participants. For visual and auditory stimulation, a TV screen was used to play videos and sounds of nature. The videos included a variety of forests, including trees and water. Olfactory stimulation was concurrently provided by placing a cotton pad with drops of cypress oil on the right shoulder of each participant. For tactile stimulation, we simulated walking on wood using an electric leg exercise machine set up with paulownia wood. The speed of the electric leg exercise machine can be adjusted to 15 steps. Based on a preliminary study, it was applied by setting the step at 2 to 3 so that it would not be a burden to the patient during hemodialysis (Table 2).

These sessions were delivered during hemodialysis. In total, 13 sessions were performed, including three 15 min sessions per week over 4 weeks, as determined by the hemodialysis schedule. The program started 3 h after hemodialysis began, as patients were expected to have the highest levels of stress, fatigue, and other physiological changes between 3 and 4 h after hemodialysis start [43]. The program was performed under the supervision of healthcare staff. 

All interventional tools in contact with the study participants were disinfected before and after application. The researcher strictly followed hand hygiene using a disinfectant before and after the intervention was applied to the participants.

### 2.4. Data Collection

This study was approved by the Human Clinical Research Ethics Committee of Kangwon National University (Approval No.: KWNUIRB-2019-12-003-001). Informed consent was obtained from the participants, and the study adhered to the Declaration of Helsinki.

Data collection took place between 13 January and 31 March 2020. Levels of emotion, fatigue, stress, and HRV of the patients in the control group were evaluated at baseline and during weeks 2 and 4. Levels of emotion, fatigue, and stress were measured by self-report. When measuring HRV, the participants were instructed to be still. Subsequently, the program was delivered to the experimental group, where the same schedule was followed. Levels of NK cells were measured at baseline and in week 4 in both groups. 

### 2.5. Statistical Analysis

Data analysis was performed using SPSS software 24.0 (IBM Corporation, Armonk, NY, USA). Homogeneity between experimental and control groups was examined using the independent *t*-test, χ^2^ test, and Fisher’s exact test. To determine program effects, a dependent variable that met the normality of distribution assumption (measure of fatigue) was tested using the repeated measures ANOVA. Other dependent variables that did not meet the normality of distribution assumption (measures of positive emotions, negative emotions, physiological stress, psychological stress, parasympathetic nervous activity, and sympathetic nervous activity) were analyzed using the generalized estimate equation. Finally, between-group differences in program effects on NK cell levels were analyzed using the independent *t*-test.

## 3. Results

### 3.1. Demographic and Clinical Characteristics of the Participants

The mean age of the experimental group was 60.35 (±2.37) years old and the mean age of the control group was 56.89 (±2.68) years, but there was no significant difference. Also, there was no significant between-group difference in demographic characteristics such as gender, marital status, education, etc. Diabetes was the most common comorbid disease in the two groups. There was no difference between the two groups in clinical characteristics including comorbidity, period of hemodialysis, hemoglobin, and albumin levels in the blood (Table 3).

Furthermore, the results of homogeneity tests for participants’ positive and negative emotion, fatigue, physiological and psychological stress, activation of sympathetic and parasympathetic nervous systems, and NK cell levels showed no significant difference between the groups at baseline (Table 4).

### 3.2. Effects of Indirect Forest Experience

**Figure 1:**
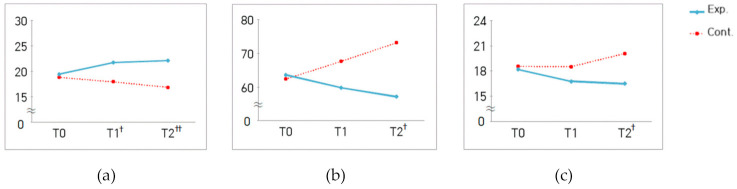
Changes to the measures of positive emotion (**a**), fatigue (**b**), and physiological stress (**c**) in both groups. T0 = baseline assessment; T1 = 2nd week of the program; T2 = 4th week of the program; ^†^ *p* < 0.05, ^††^ *p* < 0.01 indicates group differences by time point.

For measures of positive emotion, the interaction between group and measurement time was significantly different (χ^2^ = 7.26, *p* = 0.027) (Figure 1a), whereas, for measures of negative emotion, there was no significant difference in this interaction (χ^2^ = 3.63, *p* = 0.163) (Table 5). Similarly, for measures of fatigue, there was a significant interaction between group and measurement time (F = 3.75, *p* = 0.027) (Figure 1b). There was a significant difference in interaction between group and measurement time for the measures of physiological stress (χ^2^ = 9.60, *p* = 0.008) (Figure 1c), whereas no such difference was observed for the measures of psychological stress (χ^2^ = 0.84, *p* = 0.657). There was also no significant difference in interaction between group and measurement time for the activation of either parasympathetic (χ^2^ = 4.92, *p* = 0.085) or sympathetic (χ^2^ = 3.34, *p* = 0.189) nervous system (Table 5). The difference in the number of NK cells between the groups was not statistically significant (t = 0.45, *p* = 0.655) (Table 6).

## 4. Discussion

This study aimed to identify the psychological and physiological effects of an integrated indirect forest experience on hemodialysis patients’ measures of emotion, fatigue, stress, and immune function. In the present study, indirect forest experience simultaneously stimulated five senses in patients undergoing hemodialysis, providing a simulation of outdoor experiences to individuals needing to remain in a hospital. 

In our study, the measures of positive emotion increased after integrated indirect forest experience, and these changes were statistically significant. Previously, Hwang and Park [44] presented a slideshow of nature-related images to college students and found significant changes in their emotional states. Yi [45] compared urban views and nature views to establish which group of images may perform better at triggering positive emotions. In addition, Lee [37] suggested that individuals would be positively impacted if they felt the environment would give them positive effects. To summarize, integrated indirect forest experience could be applied to patients who experience emotional distress due to hemodialysis to increase their positive emotion. 

The impact of indirect forest experience on decreasing levels of fatigue has been previously reported in several studies [39,46,47]. Park [48] found psychological changes in college students exposed to the views of forests, and Jeon [23] reported that indirect forest experience may reduce the levels of tiredness. In our study, fatigue was significantly reduced in the experimental group compared to that in the control group. Hemodialysis patients are likely to experience high levels of fatigue, and indirect forest experience may be an effective fatigue management tool, provided it is continuously applied in a clinical setting.

In this study, physiological stress levels in the experimental group were significantly reduced compared those in the control group. However, psychological stress measures were not influenced by indirect forest experience. Hwang and Park’s [44] study of indirect forest visual stimulation in college students as well as that by Yi et al. [45] reported significant results. In both studies, stress was significantly reduced by indirect forest experience. Since the participants of our study were patients with chronic renal failure, it is expected that there will be differences in psychological stress among university students. In particular, in the present study, the levels of psychological stress were higher than those of physiological stress, and this finding is consistent with that of Kim and Yang’s [4] studies, whereby hemodialysis patients had high levels of psychosocial stress. 

In the present study, there was no significant difference in either parasympathetic or sympathetic activity between pre- and post-intervention, as measured by HRV. Previously, Alvarsson et al. [49] reported that parasympathetic activity was not influenced by auditory stimulation with forest-associated sounds. However, Igarashi et al. [50] and Ikei et al. [51] have shown that parasympathetic activity was affected by olfactory and tactile stimulation respectively, resulting in physiological relaxation. A study by Jo et al. [52] has shown a significant decrease in stress levels as a result of decreased activation of the sympathetic nervous system. Considering the discrepancies in previous study findings, further research is required to determine the effect of indirect forest therapy types on nervous system activity. The reason why it is not significant in HRV may be related to an abnormal autonomic function. The majority of patients receiving hemodialysis have diabetes. One of the most overlooked serious complications of diabetes is cardiovascular autonomic neuropathy. In addition, if blood sugar is not controlled, HRV may be low owing to abnormal autonomic function [53]. In our study, about 40% of the participants had diabetes. Playing a key part in the immune system, NK cells are sensitive to stress [21], and higher levels of stress may reduce the activity of NK cells [54]. In our study, integrated indirect forest experience did not affect NK cell levels. This result was different from that of a previous study, which reported higher levels of NK cell activation in a forest meditation group than in the control group [22]. In addition, a 12-week high-intensity forest walking intervention has been previously reported to significantly increase the number of NK cells [55]. A study that applied a three-day bamboo forest therapy in male students also reported that their NK cell levels increased following this intervention [56]. Since our study applied indirect forest experience for 4 weeks, its effects are likely to be different from those previously reported. Given that NK cells activation in hemodialysis patients tends to be lower than that in healthy individuals [57], it is advisable to apply the present program over a longer period and to re-examine its effect on immunity. There are many confounding factors, such as the filter type of the hemodialysis machine [58] and the period of dialysis [59], which might influence the NK cell levels in the dialysis population. Further study should be conducted considering these confounding factors to improve immune function by applying forest experience in hemodialysis patients. 

Age and education level are factors affecting the needs of hemodialysis patients [60]. Younger people need extensive psychological assistance to cope with negative emotion related to the disease. Additionally, young hemodialysis patients complained of more lack of energy, mobility limitations, and sleep disturbances than elderly hemodialysis patients [61]. Therefore, indirect forest experience may be recommended to young patients during hemodialysis to improve emotion, fatigue, and stress. The participants of this study were relatively young, with an average age of 58 years, and they had a high level of education, so they actively participated in indirect forest experience. With the active participation of the experimental group, positive emotion, fatigue, and physiological stress steadily improved over the second and fourth weeks of intervention, whereas the values of the control group appeared to be unchanged or worsened. Furthermore, participants in the experimental group reported that the program helped them feel like the hemodialysis session went faster than usual, and they were satisfied with the experience of “being in nature.” Because most of the patients have to undergo hemodialysis for a lifetime, indirect forest experience can be recommended as an active strategy to improve their positive emotion, fatigue, and physiological stress.

This study had several limitations. First, it was difficult to control the impact of other environmental conditions, including the sounds of hemodialysis machines and other medical equipment, which may have affected the presented findings. Second, the participants were not randomly assigned to the experimental and control groups. Randomized controlled trials should be conducted in the future. Third, hemodialysis patients find it difficult to remain still while undergoing hemodialysis, resulting in movements that may have influenced the HRV values. Fourth, immune function was measured with a single index, specifically, NK cell levels. Future studies should use other indicators to examine the impact of indirect forest therapy on the immune function.

## 5. Conclusions

Indirect forest experience may promote positive emotions and reduce the levels of fatigue and stress in patients undergoing hemodialysis. This finding suggests that indirect forest experience may be useful to patients undergoing hemodialysis and those undergoing other forms of long-term treatment, for example, chemotherapy. This intervention may also help patients relax and rest during their treatment.

## Figures and Tables

**Table 1:** Zuckerman inventory of personal reaction scale (ZIPERS) subclass and questions.

Subclass	Questions
Positive affect (4)	I felt carefree or playful
	I felt affectionate or warmhearted
	I felt elated or pleased
	I felt like acting friendly or affectionate
Attentiveness (2)	I felt attentive or in deep thought
	I felt like getting out of this situation or avoiding it (reverse)
Fear (3)	My heart was beating fast
	I was breathing fast
	I felt fearful
Sadness (1)	I felt sad
Anger (2)	I felt angry or defiant
	I felt angry or acted aggressively

**Table 2:** Five types of indirect forest experience.

Categories	Contents
Visual stimuli	Nature videos provided by TV monitor
Auditory stimuli	Sounds of nature (water, wind, insects, birds, etc.)
Olfactory stimuli	Cotton pad with drops of cypress oil placed
Tactile stimuli	Paulownia scaffold applied
Motor stimuli	Electric leg exercise machine for bed applied in 2 to 3 steps

**Table 3:** Demographic and clinical characteristics of the groups (*n* = 54).

Characteristics	Categories	Exp. (*n* = 26)	Cont. (*n* = 28)	χ^2^ or z	*p*
		Mean ± SD/*n*(%)	Mean ± SD/*n*(%)		
Age (year)		60.35 ± 2.37	56.89 ± 2.68	27.63	0.640
Gender	Male	18(69.2)	22(78.6)	0.61	0.434
	Female	8(30.8)	6(21.4)		
Marital status	Married	23(88.5)	22(78.6)	0.95	0.470 ^†^
	Single	3(11.5)	6(21.4)		
Education	<Middle	8(30.8)	6(21.4)	0.79	0.675
	High	7(26.9)	10(35.7)		
	University	11(42.3)	12(42.9)		
Occupation	Yes	12(46.2)	11(39.3)	0.26	0.610
	No	14(53.8)	18(60.7)		
Religion	Yes	16(61.5)	18(64.3)	0.04	0.835
	No	10(38.5)	10(35.7)		
Comorbidity	Diabetes	12(46.2)	10(35.7)	1.25	0.535
	Hypertension	9(34.6)	9(32.1)		
	etc.	5(19.2)	9(32.1)		
Period of hemodialysis (year)	<5	16(61.5)	14(50.0)	0.73	0.394
	≥5	10(38.5)	14(50.0)		
Vascular state for hemodialysis	Fistula	23(88.5)	25(89.3)	0.01	>0.999 ^†^
	Graft	3(11.5)	3(10.7)		
Hemodialysis vessel location	Upper arm	9(34.6)	7(25.0)	0.60	0.439
	Forearm	17(65.4)	21(75.0)		
Hemoglobin (g/dl)		10.77 ± 1.19	10.73 ± 0.81	30.84	0.324
Albumin (g/dl)		4.02 ± 0.21	4.00 ± 0.33	12.50	0.406

Cont. = control group; Exp. = experimental group; SD = standard deviation; ^†^ Fisher’s exact test.

**Table 4:** Values of dependent variables for the groups (*n* = 54).

Variables		Exp. (*n* = 26)	Cont. (*n* = 28)	t	*p*
		Mean ± SD	Mean ± SD		
Emotion	Positive emotion	19.46 ± 6.24	18.82 ± 6.15	−0.38	0.706
	Negative emotion	4.89 ± 7.80	4.36 ± 4.79	−0.30	0.764
Fatigue		63.62 ± 28.14	62.39 ± 27.58	−0.16	0.873
Stress	Physiological stress	18.19 ± 5.84	18.57 ± 5.74	0.24	0.811
	Psychological stress	21.85 ± 7.42	21.14 ± 5.90	−0.39	0.701
HRV	High frequency (HF)	6.48 ± 0.71	6.45 ± 0.96	−0.15	0.883
	Low frequency (LF)	6.76 ± 0.34	6.72 ± 0.54	−0.32	0.747
NK cells (%)	CD16 + CD56	11.80 ± 6.64	14.24 ± 9.82	1.06	0.295

Cont. = control group; Exp. = experimental group; HRV = heart rate variability; NK cells = natural killer cells; SD = standard deviation.

**Table 5:** Effect of indirect forest experience on emotion, fatigue, stress, and HRV (*n* = 54).

Variables			Exp. (*n* = 26)	Cont. (*n* = 28)	Source	F or χ^2^	*p*
			Mean ± SD	Mean ± SD			
Emotion	Positive emotion	T0	19.46 ± 6.24	18.82 ± 6.15	Group	5.74	0.017
		T1	21.73 ± 6.02	17.93 ± 6.06	Time	0.87	0.648
		T2	22.12 ± 5.19	16.86 ± 5.84	Group-by -time interaction	7.26	0.027 ^†^
	Negative emotion	T0	4.89 ± 7.80	4.36 ± 4.79	Group	2.12	0.145
		T1	3.73 ± 6.42	4.61 ± 5.38	Time	0.22	0.898
		T2	3.54 ± 5.44	5.68 ± 6.42	Group-by -time interaction	3.63	0.163 ^†^
Fatigue		T0	63.62 ± 28.14	62.39 ± 27.58	Group	1.35	0.251
		T1	59.81 ± 22.07	67.61 ± 27.80	Time	0.24	0.788
		T2	57.15 ± 25.63	73.11 ± 31.04	Group-by -time interaction	3.75	0.027
Stress	Physiological stress	T0	18.19 ± 5.84	18.57 ± 5.74	Group	1.87	0.171
		T1	16.77 ± 5.54	18.54 ± 5.20	Time	2.97	0.227
		T2	16.50 ± 5.18	20.11 ± 6.46	Group-by -time interaction	9.60	0.008 ^†^
	Psychological stress	T0	21.85 ± 7.42	21.14 ± 5.90	Group	0.30	0.584
		T1	21.58 ± 5.99	21.11 ± 6.29	Time	0.35	0.838
		T2	21.85 ± 6.65	20.39 ± 6.81	Group-by -time interaction	0.84	0.657 ^†^
HRV	High frequency	T0	6.48 ± 0.71	6.45 ± 0.96	Group	1.26	0.261
		T1	6.94 ± 1.09	6.41 ± 0.90	Time	5.12	0.077
		T2	6.55 ± 0.95	6.36 ± 0.94	Group-by -time interaction	4.92	0.085 ^†^
	Low frequency	T0	6.76 ± 0.34	6.72 ± 0.54	Group	0.81	0.368
		T1	6.94 ± 0.49	6.73 ± 0.51	Time	3.30	0.192
		T2	6.79 ± 0.46	6.73 ± 0.53	Group-by -time interaction	3.34	0.189 ^†^

Cont. = control group; Exp. = experimental group; HRV = heart rate variability; SD = standard deviation; T0 = baseline assessment; T1 = 2nd week of the program; T2 = 4th week of the program; ^†^ generalized estimate equation.

**Table 6:** Effect of indirect forest stimulation on NK cell levels (*n* = 54).

Variables	Group	Pretest	Posttest	Mean Difference	t	*p*
		Mean ± SD	Mean ± SD	Mean ± SD		
NK cells (%)	Exp. (*n* = 26)	11.80 ± 6.64	11.18 ± 5.94	−0.62 ± 2.09	0.45	0.655
	Cont. (*n* = 28)	14.24 ± 9.82	13.21 ± 8.82	−1.02 ± 4.09		

Cont. = control group; Exp. = experimental group; NK cells = natural killer cells; SD = standard deviation.

## Data Availability

Not applicable.
